# Modulation of Vaccine-Induced HIV-1-Specific Immune Responses by Co-Electroporation of PD-L1 Encoding DNA

**DOI:** 10.3390/vaccines8010027

**Published:** 2020-01-14

**Authors:** Pierre Tannig, Antonia Sophia Peter, Dennis Lapuente, Stephan Klessing, Dominik Damm, Matthias Tenbusch, Klaus Überla, Vladimir Temchura

**Affiliations:** Institute of Clinical and Molecular Virology, Friedrich-Alexander-University Erlangen-Nürnberg, 91054 Erlangen, Germany; pierre.tannig@fau.de (P.T.); antonia.sophia.peter@uk-erlangen.de (A.S.P.); dennis.lapuente@uk-erlangen.de (D.L.); stephan.klessing@uk-erlangen.de (S.K.); dominik.damm@uk-erlangen.de (D.D.); matthias.tenbusch@fau.de (M.T.); klaus.ueberla@fau.de (K.Ü.)

**Keywords:** HIV-1, checkpoint inhibitors, checkpoint blockade, intramuscular electroporation, soluble immune checkpoints, immunomodulation

## Abstract

The importance of a balanced T_H_1/T_H_2 humoral immune response against the HIV-1 envelope protein (Env) for antibody-mediated HIV-1 control is increasingly recognized. However, there is no defined vaccination strategy to raise it. Since immune checkpoints are involved in the induction of adoptive immunity and their inhibitors (monoclonal antibodies) are licensed for cancer therapy, we investigated the effect of checkpoint blockade after HIV-1 genetic vaccination on enhancement and modulation of antiviral antibody responses. By intraperitoneal administration of checkpoint antibodies in mice we observed an induction of anti-drug antibodies which may interfere with immunomodulation by checkpoint inhibitors. Therefore, we blocked immune checkpoints locally by co-electroporation of DNA vaccines encoding the active soluble ectodomains of programmed cell death protein-1 (PD-1) or its ligand (PD-L1), respectively. Plasmid-encoded immune checkpoints did not elicit a detectable antibody response, suggesting no interference with their immunomodulatory effects. Co-electroporation of a HIV-1 DNA vaccine formulation with soluble PD-L1 ectodomain increased HIV-1 Env-specific T_H_1 CD4 T cell and IgG2a antibody responses. The overall antibody response was hereby shifted towards a more T_H_1/T_H_2 balanced subtype pattern. These findings indicate that co-electroporation of soluble checkpoint ectodomains together with DNA-based vaccines has modulatory effects on vaccine-induced immune responses that could improve vaccine efficacies.

## 1. Introduction

Even after more than 30 years of vaccine research, the development of a prophylactic HIV-1 vaccine is still facing serious obstacles. Efficacy trials data on immune responses that correlate with reduced risk of HIV-1 infection are limited [1]. In the RV144 Thai trial, an envelope protein (Env) subunit vaccination regiment resulted in a moderate efficacy of 31.2% [2]. The elicitation of IgG3 antibodies targeting the variable regions 1 and 2 (V1V2) of Env was identified as correlate of protection, raising interest in antibody-mediated effector functions [3,4,5]. Env-specific IgG3 antibodies (IgG subclass in humans that is associated with T_H_1-response [6]) further showed an enhanced virion internalization activity in monocytes compared to other antibody isotypes [7]. These data demonstrate that the antibody subtype pattern elicited after vaccination may play a crucial role in mediating antibody-directed effector functions eventually resulting in viral clearance.

Intramuscular electroporation (i.m. EP) as a measure to induce strong cellular and humoral immune responses has been used for a variety of antigens [8]. In non-human primates, we demonstrated that i.m. EP delivery of vaccine DNA encoding for the fusion protein of respiratory syncytial virus (RSV) [9] or p27 capsid protein of simian immunodeficiency virus (SIV) [10] led to substantially higher antibody responses compared to the conventional i.m. DNA immunization. However, in contrast to the surface proteins of RSV and Influenza A, i.m. EP of plasmids encoding for HIV-1 Env elicits an antibody response strongly biased towards the T_H_2-associated IgG1 subclass in mice [11,12].

Immune checkpoints are a class of molecules capable of enhancing or inhibiting T cell signaling cascades in order to guarantee immune tolerance and control of inflammation [13,14]. The most prominent members of this group are the programmed cell death protein-1 (PD-1) and its ligands PD-L1 and PD-L2. Targeting those proteins with monoclonal antibodies can overcome T cell exhaustion and restore T cell functions in the tumor microenvironment [15]. Checkpoint inhibitors (CPI) directed against PD-1 and its ligands have been established as a platform of immunomodulation and are broadly used in the treatment of melanoma and other cancers [16,17].

During antigen-mediated immune responses PD-1 controls follicular T-helper cell positioning and function during germinal center reactions [18] and regulates germinal center B cell survival, affinity maturation, and formation of long-lived plasma cells [19]. However, the applicability of checkpoint blockade for modulation of immune responses induced by prophylactic vaccinations has not been thoroughly investigated.

In this study, we investigated whether different checkpoint inhibitors have a modulatory effect on the vaccine-induced HIV-1-specific immune responses. Therefore, we blocked immune checkpoints either systemically by monoclonal antibody administration or locally by electroporation of DNA encoding for the soluble ectodomains of PD-1 or PD-L1 in mice receiving anti-HIV-1 immunization.

## 2. Materials and Methods

### 2.1. Mice Housing, Immunizations, and Ethics Statement

Five- to six-week old BALB/c mice were purchased from Charles River Laboratories (Wilmington, USA) and housed in individual ventilated cages in accordance with the national law and institutional guidelines at the Franz-Penzoldt-Center of the Faculty of Medicine, University Clinics Erlangen (Erlangen, Germany) and at the animal facility of the Faculty of Medicine, Ruhr University Bochum (Bochum, Germany). A total of 207 mice were used for the study and the data of 205 animals are shown. Two died due to anesthesia-related circumstances.

For DNA immunizations, mice were anesthetized by continuous inhalation with isoflurane (CP-Pharma, Burgdorf, Germany). Hind legs were shaved and 2.5-mm electrode spacing bearing the centered injection needle from the TRiGrid electrode array (Ichor Medical, San Diego, CA, USA) were applied. A total of 30–45 µg total plasmid DNA in a total volume of 60 µL PBS was injected i.m. in each hind leg. Electrical Signals of 63 V amplitude and 40 mS duration were immediately applied after injection.

For in vivo blockade of immune checkpoints, 200 µg of in vivo grade monoclonal antibodies (all from BioXCell, West Lebanon, NH, USA) against PD-1 (J43, Armenian Hamster IgG), PD-L1 (10F.9G2, Rat IgG2b), PD-L2 (TY25, Rat IgG2a), or isotype control (anti-KLH, LTF-2, Rat IgG2b) were injected intraperitoneally starting two days after immunization in three-day intervals over a total time-period of two weeks.

All conducted animal experiments were approved by the Government of Lower Franconia according to the license 55.2-2532-2-203 and by an external ethics committee authorized by the North Rhine-Westphalia State Office for Consumer Protection and Food Safety (license 84-02.04.2013-A371).

### 2.2. Plasmids

The codon-optimized HIV-1 expression plasmids Hgpsyn [20] encoding for gag/pol and pConBgp140GC/D [21] encoding for a clade B envelope protein carrying the cytoplasmic domain of VSV-G were used for virus-like particle (VLP) production and DNA immunization. The luciferase-encoding plasmid pLuc-empty was used for analysis of antigen expression in vivo. The expression plasmids encoding for either the soluble ectodomains of PD-1 or PD-L1 were used as DNA vectors. The pVax vector system (Invitrogen, Carlsbad, CA, USA) was used as mock control.

### 2.3. Analysis of Antigen Expression In Vivo

Mice were electroporated intramuscularly with 20 µg luciferase-encoding plasmid. At the indicated time points after immunization, 200 µg d-luciferin was injected into both hind legs under light anesthesia by inhalation of isoflurane. Luminescence signals were measured 3 min later with an IVIS Lumina Series II (PerkinElmer, Waltham, MA, USA). The signals were quantified in the red-circled areas for both mice at all four time points. Background luminescence signals are shown as well (ROI 5).

### 2.4. Analysis of Humoral Immune Responses

In order to monitor the humoral immune responses, mice were bled at different time-points after immunization by puncture of the retro orbital sinux with a heparinized capillary (Hirschmann Laborgeräte, Eberstadt, Germany). After centrifugation for 5 min at 2370× *g*, sera were stored at −20 °C until further use. Antigen-specific antibody production was determined by a gp120 ELISA. A quantitative assessment of serum antibody titers was performed by using the gp120-specific antibody b12 fused to the murine heavy chains IgG1 or IgG2a (Figure S1). Then, 96-well microtest plates (Sarstedt, Nümbrecht, Germany) were coated with 100 ng of pConBgp120-His in bicarbonate buffer (pH 9.6) at room temperature overnight. To determine antibody responses against the CPIs, plates were coated with the respective treatment antibodies (100 ng/well). After washing the plates with PBS-T, wells were blocked with 5% skimmed milk in PBS-T following an additional washing step. Incubation with diluted sera was performed in 2% skimmed milk. The respective antibody subtypes were detected after a final washing step by the HRP-conjugated antibodies directed against IgG1, IgG2a, IgG2b, and IgG3 (Southern Biotech, Birmingham, AL, USA). Finally, the plates were washed, and relative light units were detected with the multilabel plate reader Victor (Perkin Elmer, Hamburg, Germany).

### 2.5. Analysis of Cellular Immune Responses

HIV-1 Env-specific T cell responses in the spleens were detected by intracellular cytokine staining (ICS) as previously described [12]. Briefly, mice were sacrificed, spleens removed, and single-cell suspensions prepared by homogenization through a 70 µm cell strainer (Corning Inc., Corning, Harrodsburg, KY, USA). After erythrocyte lysis, splenocytes were resuspended in RPMI 1640 (Gibco, ThermoFisher Scientific, Waltham, MA, USA) supplemented with 10% FCS (Sigma Aldrich, Taufkirchen, Germany), 1% penicillin/streptomycin (Sigma Aldrich, Taufkirchen, Germany), 10 mmol HEPES (Gibco, ThermoFisher Scientific, Waltham, MA, USA), 2 mmol L-glutamine (Gibco, ThermoFisher Scientific, Waltham, MA, USA), and 50 µmol β-Mercaptoethanol (PAN-Biotech, Aidenbach, Germany).

For the ICS, 10^6^ splenocytes/well were seeded in a 96-well U-bottom microtiter plate (Greiner Bio-One, Frickenhausen, Germany) and stimulated with 5 μg/mL of the MHC-II–restricted peptides GVPVWKEATTTLFCASDAKA for HIV-1 Env or a mixture of SPEVIPMFSALSEGA and PVGEIYKRWIILGLN for HIV-1 Gag in the presence of 2 μg/mL anti-CD28 (37.51; eBioscience, Frankfurt am Main, Germany) and 3 μg/mL Brefeldin A (eBioscience, Frankfurt am Main, Germany) for 6 h at 37 °C in a humidified 5% CO_2_ atmosphere. After stimulation, the cells were stained with anti-mouse CD4 BV650 (RM4-5, Biolegend, San Diego, CA, USA) and Fixable Viability Dye eFluor 450 (eBioscience, Frankfurt am Main, Germany). Afterwards, cells were fixed with 2% paraformaldehyde and permeabilized with 0.5% saponin (Sigma Aldrich, Taufkirchen, Germany) in the presence of 1.7 µg/mL anti-mouse CD16/CD32 (93; eBioscience). Intracellular cytokine staining was performed in 0.5% saponin using anti-mouse TNFα PE-Cy7 (MP6-XT22), anti-mouse IL-2 APC (JES6-5H4), and anti-mouse IFNγ PE (XMG1.2, all from eBioscience). Samples were measured on the FACS-LSR II (BD, Franklin Lakes) and data was analyzed using FlowJo (Tree Star, Ashland, OR, USA). For detection of IL-4 and IL-5 by ELISA, 10^6^ splenocytes/well were incubated in the presence of 2 µg/mL anti-CD28 (37.51; Life Technologies, Carlsbad, CA, USA) for 72 h at 37 °C in a humidified 5% CO_2_ atmosphere. To determine antigen-specific production of cytokines, previously mentioned HIV-1 Env and Gag peptides were included into the incubation mixture. Supernatants were diluted and IL-4 and IL-5 concentration analyzed by Ready-SET-Go ELISA (Life Technologies, Carlsbad, CA, USA) according to the manufacturer’s protocol.

### 2.6. Staining of Regulatory T Cells

For the T cell staining, 10^6^ splenocytes/well were seeded in 96-well U-bottom plates and surface stained with anti-mouse CD4 BV650 (RM4-5, Biolegend, San Diego, CA, USA), anti-mouse CD25 APC (PC61, BD Pharmingen, San Jose, CA, USA), and Fixable Viability Dye eFluor450 (eBioscience). After fixation and permeabilization, splenocytes were stained intracellularly with anti-mouse Foxp3 PE (MF14, Biolegend, San Diego, CA, USA). Samples were measured on the FACS-LSR II (BD, Franklin Lakes, NJ, USA) and data was analyzed using FlowJo (Tree Star, Ashland, OR, USA).

### 2.7. Cell Culture

Human embryonic kidney cell line 293T (HEK 293T, obtained from European Collection of Cell Cultures, Salisbury, UK) were maintained in DMEM (Gibco, ThermoFisher Scientific, Waltham, MA, USA) supplemented with 10% FCS (Sigma Aldrich, Taufkirchen, Germany), 1% penicillin/streptomycin (Sigma Aldrich, Taufkirchen, Germany), and 2 mM L-glutamine (Gibco, ThermoFisher Scientific, Waltham, MA, USA)).

FreeStyle 293F (obtained from Thermo Fisher, Schwerte, Germany) cells were maintained stirring as recommended by the manufacturer in a humidified 8% CO_2_ atmosphere. The cells were cultured in a density between 0.5 and 2 × 10^6^ cells/mL.

### 2.8. Protein Production and Purification

FreeStyle 293F cells were transfected with 80 µg of expression plasmids encoding for soluble pConBgp120-His in sterile disposable PETG flasks (Wagner and Munz GmbH, Munich, Germany) with 3 µg polyethylenimine (Sigma Aldrich, Taufkirchen, Germany) per 1 µg DNA. The transfection mix was prepared in OPTI-MEM Reduced Medium (Thermo Fisher, Schwerte, Germany). Medium was changed 6 h after transfection. Three days post-transfection, supernatants were collected and sterile-filtered through 0.2 µm Minisart filters (Sigma Aldrich, Taufkirchen, Germany) and purified via lectin affinity chromatography using lectin from *Galanthus nivalis* (Vector Laboratories Inc., Burlingame, CA, USA). Columns were loaded after washing with PBS containing 1 mM EDTA and 1 mM EGTA (both Sigma Aldrich, Taufkirchen, Germany). After loading, columns were washed and protein eluted using a 19.5% solution of Methy-α-d-mannopyranosid (Merck, Darmstadt, Germany). Carbohydrates in the eluate were dialyzed. The purified protein was concentrated over Amicon Centrifugal Filters with 10 kDa cut-off (Merck, Darmstadt, Germany). Protein concentration was measured using the ND100-NanoDrop^®^ (peQlab, Erlangen, Germany). Samples were stored at 4 °C until further use.

### 2.9. VLP Preparation and Quantification

293T cells were transfected with each 40 µg of the expression plasmids encoding for pConBgp140-GCD and Hgpsyn in 175-cm^2^ flasks (Greiner Bio One, Frickenhausen, Germany) with 1.25 µg polyethylenimine (Sigma Aldrich, Taufkirchen, Germany) per 1 µg DNA. Two days post-transfection, VLPs in the supernatant were purified by ultracentrifugation through a 35% sucrose cushion at 133.900× *g* and 4 °C for 2.5 h. VLPs were resuspended in sterile PBS and stored at −80 °C until further use.

HIV-1 Env and Gag concentration in the VLP preparations were quantified by ELISA. For that, different VLP dilutions together with a dilution series of pConSgp140 (Polymun Scientific, Klosterneuburg, Austria) and p24 (Aalto Bio Reagents, Dublin, Ireland) were coated in bicarbonate buffer (pH 9.6) on 96-well microtest plates (Sarstedt, Nümbrecht, Germany) at RT overnight. After washing the plates with PBS-T, wells were blocked with 5% skimmed milk in PBS-T followed by an additional washing step. Incubation with the HIV-1 Env antibody 2G12 or the anti-p24 antibody (produced in hybridoma cells) was performed in 2% skimmed milk. After washing, HRP-conjugated antibodies directed against human or mouse IgG (Dianova, Hamburg, Germany) were added. Finally, plates were washed and relative light units (RLUs) were detected with the multilabel plate reader Victor (Perkin Elmer, Hamburg, Germany).

Virus-like particle size and PDI were analyzed using the ZetaSizer Nano S90 (Malvern Pananalytical, Kassel, Germany) (Figure S2).

### 2.10. Statistical Analysis

Data are presented as means ± standard errors of the means (SEM). In the figure legends, *n* = X refers to the used animals per group. Statistical analysis was performed as indicated in figure legends with GraphPad Prism software version 7 (Graphpad Software Inc., San Diego, CA, USA) using one-way analysis of variance (ANOVA) with Tukey’s post-test or unpaired *t* tests.

## 3. Results

### 3.1. Checkpoint Inhibition by Monoclonal Antibodies after VLP Immunization

**Figure 1:**
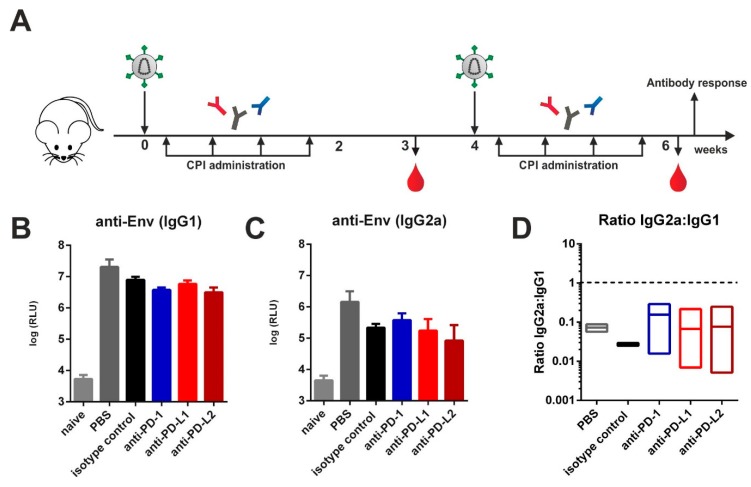
Checkpoint inhibition by monoclonal antibody administration after virus-like particle (VLP) immunization. (**A**) Six-week old BALB/c mice were intramuscularly immunized with VLPs containing Env and Gag. Two days after immunization, 200 µg of checkpoint inhibitors (CPIs) or isotype control were administered intraperitoneally in three-day intervals over a total time-period of two weeks. Animals received a booster immunization with a follow-up CPI administration that was identical to the priming regimen. Blood was drawn at weeks 3 and 6 and antibody responses analyzed. Env-specific IgG1 (**B**) and IgG2a (**C**) antibody responses and IgG2a:IgG1 ratios (**D**) in the sera of BALB/c mice six weeks after priming. Shown are mean values with standard errors of the means (SEM) (*n* = 4 for experimental groups, *n* = 2 for controls (naïve, PBS)).

Previously we reported that immunization of mice against HIV-1 Env with both protein and DNA vaccines induces a T_H_2-associated immune response leading to IgG1 Env-specific Ab responses with reduced effector functions [11,12,22]. Here we investigated whether this pattern might be switched to the T_H_1-associated IgG2a subclass by blocking immune checkpoints. For that, VLPs containing HIV-1 Gag and Env were injected intramuscularly in a prime-boost regimen into naïve BALB/c mice. Two days after each immunization, mice were treated with either PBS, an isotype control, or monoclonal antibodies directed against PD-1 or its ligands PD-L1 and PD-L2 according to published protocols (Figure 1A) [23,24]. After the boosting immunization, however, we observed no significant differences regarding the levels of IgG1 (Figure 1B) and IgG2a (Figure 1C) between all experimental groups immunized with VLPs. The IgG1 to IgG2a ratios also remained unaffected (Figure 1D).

### 3.2. Checkpoint Inhibition by Monoclonal Antibodies after DNA Immunization

**Figure 2:**
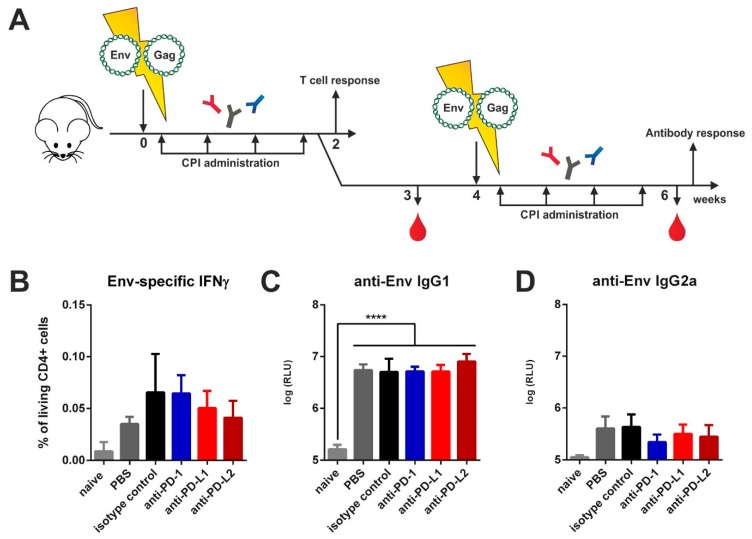
Checkpoint inhibition by monoclonal antibodies after DNA electroporation. (**A**) Six-week old BALB/c mice were electroporated intramuscularly with expression plasmids encoding for Env and Gag. Two days after electroporation, 200 µg of CPIs or isotype control were administered via the intraperitoneal route in three-day intervals over a total time-period of two weeks. After two weeks, half of the animals were sacrificed, and T cell responses analyzed. The other animals received a booster immunization with a follow-up CPI administration identical to the priming regimen. Blood was drawn at weeks 3 and 6 and antibody responses analyzed. (**B**) Percentage of CD4+ T cells producing IFNγ after in vitro stimulation with HIV Env T helper peptide (measured by intracellular cytokine staining). Shown are mean values with SEM (*n *= 4). Env-specific IgG1 (**C**) and IgG2a (**D**) antibody responses in the sera of BALB/c mice six weeks after priming. Shown are mean values with SEM (*n* = 4–6) and significant differences between the groups (one-way ANOVA analyses followed by Tukey’s multiple comparison test; **** *p* < 0.0001).

In order to provide antigen expression over the time-course of checkpoint blockade, we electroporated mice intramuscularly with plasmids encoding for Env and Gag. After electroporation, an identical CPI administration regimen was conducted as in the VLP immunized animals (Figure 2A). Since DNA immunization elicits strong cellular responses, we first analyzed antigen-specific T cell responses two weeks after priming by ICS. For Env-specific CD4 T cells no differences in IFNγ (Figure 2B), IL-2 (Figure S3), and TNFα (Figure S3) cytokine production after in vitro re-stimulation were detected. The frequency of polyfunctional T cells secreting those cytokines simultaneously remained unaffected as well (Figure S3). By measuring the antibody responses two weeks after boosting we detected a strong induction of IgG1 antibodies in all immunized groups but no differences between the isotype- and CPI-treated groups (Figure 2C). For T_H_1-associated Env-specific IgG2a antibodies also no difference was observed (Figure 2D). After Gag-specific re-stimulation however, we observed a significantly upregulated production of IL-2 by CD4 T cells in the animals treated with the anti-PD-L2 antibody (Figure S4A). However, the Gag-specific IgG1 and IgG2a antibodies remained unaffected after checkpoint blockade (Figure S4B,C).

Thus, in our experiments commonly employed checkpoint inhibition with monoclonal antibodies had no effect on HIV-1 Env specific antibody responses induced by protein and DNA vaccines.

### 3.3. Checkpoint Inhibition with Monoclonal Antibodies Induces Anti-Drug Responses

**Figure 3:**
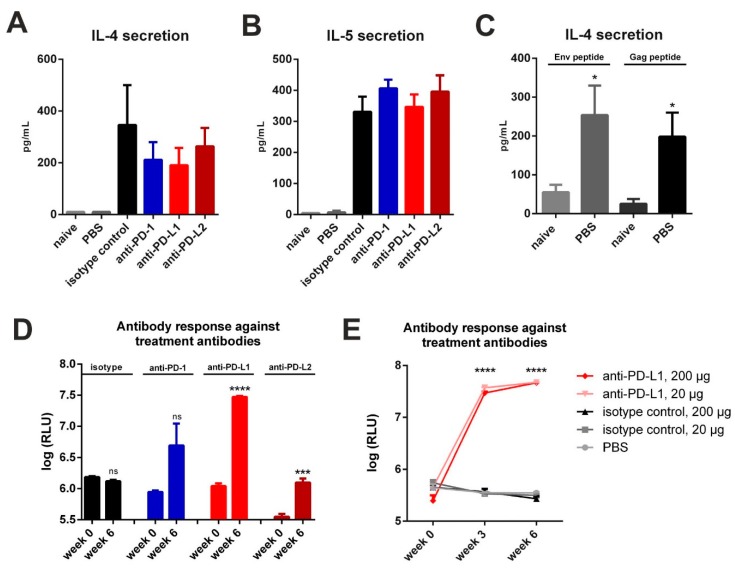
Checkpoint inhibition by monoclonal antibodies elicits a competitive immune response. Spontaneous secretion of IL-4 (**A**) and IL-5 (**B**) after three-day culture of splenocytes of BALB/c mice two weeks after immunization and two days after the last CPI treatment. Shown are mean values with SEM (*n* = 2–4). (**C**) Antigen-specific IL-4 secretion in naïve and immunized mice receiving PBS treatment. Shown are mean values, SEM (*n* = 5) and significant differences between groups (unpaired *t*-test, * *p* < 0.05). (**D**) Auto-antibody responses with 200 µg CPI administration in three-day intervals after prime-booster DNA immunization regimens. The plates were coated with respective treatment antibodies. Shown are mean values with SEM (*n *= 3–4) and significant differences between time-points (unpaired *t*-test, ns = not significant, *** *p* < 0.001, **** *p *< 0.0001). (**E**) Auto-antibody response with 20 and 200 µg CPI administration in three-day intervals after prime-booster VLP immunization regimens. The plates were coated with respective treatment antibodies. Shown are mean values with SEM (*n* = 3–4) and significant differences between groups receiving anti-PD-L1 (programmed death-ligand 1) treatment against isotype control and PBS groups (two-way ANOVA analyses followed by Tukey’s multiple comparison test; **** *p* < 0.0001).

Since used monoclonal antibodies contained large xenogeneic Fc-domains (Armenian Hamster IgG for anti-PD-1, Rat IgG2b for anti-PD-L1, Rat IgG2a for anti-PD-L2), repetitively administrated, they may induce host immune response against these treatment antibodies [25]. Additionally, peritoneal macrophages and other cells of innate immunity might react on xenogeneic proteins and promote T_H_2-assosiated responses [26]. Therefore, we first performed a three-day incubation of splenocytes from DNA-primed and CPI treated mice (Figure 2A) in the presence of co-stimulatory anti-CD28 and checked for IL-4 and IL-5 secretion. In DNA-immunized animals treated only with PBS after electroporation, there was a minimal basal secretion of these cytokines detectable (Figure 3A,B). However, in vitro re-stimulation of this group with MHC-II restricted Env and Gag peptides resulted in an antigen-specific cytokine production (Figure 3C). In contrast, in all immunized mice that were treated with an antibody, we observed a substantial secretion of the T_H_2 cytokines IL-4 and IL-5 in the absence of antigenic stimulation (Figure 3A,B). Additional antigen-specific re-stimulation of these cells did not significantly alter these elevated levels of IL-4 and IL-5 production (Figure S5).

Next, we checked for the induction of humoral immune responses directed against the monoclonal antibodies to checkpoint molecules by ELISA. Here we observed an induction of anti-drug IgG1 antibodies in all CPI-treated groups with anti-PD-L1 treated animals showing the highest antibody production (Figure 3D). In the animals which received the isotype control, no humoral response was observed. We also confirmed this observation for anti-PD-L1 and isotype treatment antibodies in the VLP vaccine-delivery system (Figure 3E). In order to elucidate whether the response can be prevented by using reduced amounts of the anti-PD-L1 antibodies, we performed a VLP prime-boost experiment and treated mice with 20 µg (10-fold reduction) of anti-PD-L1 or the isotype control. However, the dosage reduction had no effect on the IgG1 response against anti-PD-L1 antibodies. Taken together, the intraperitoneal application of commercially available monoclonal antibodies for checkpoint inhibition induced anti-drug immune responses in mice.

### 3.4. Co-Electroporation of DNA Encoding Soluble PD-1 and PD-L1 Ectodomains Does Not Induce Anti-Drug Antibodies

**Figure 4:**
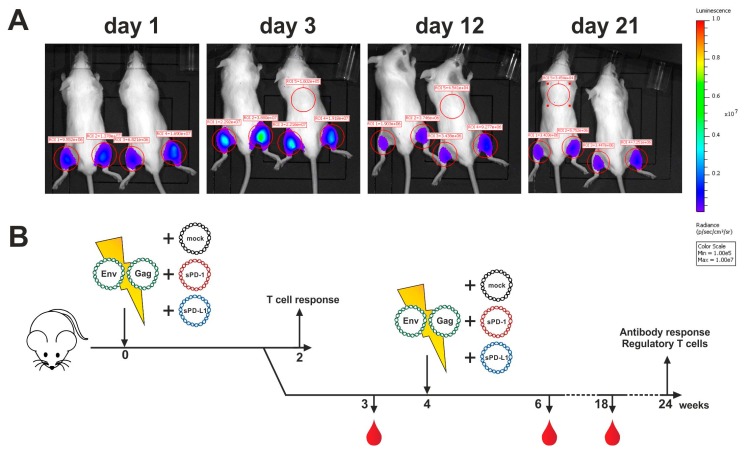
Immunization outline with soluble ectodomain co-expression. (**A**) Long-term antigen expression after DNA electroporation. Two BALB/c mice were intramuscularly electroporated with 20 µg luciferase-encoding plasmid. Luminescence signals were quantified in the red-circled areas for both mice at indicated time-points after electroporation. Background luminescence signals are shown as well (ROI 5). (**B**) Six-week old BALB/c mice were electroporated intramuscularly with expression plasmids encoding for Env and Gag. Additionally, the animals were either co-electroporated with an empty vector (mock) or plasmids encoding for the soluble ectodomains of PD-1 (sPD-1) or PD-L1 (sPD-L1). After two weeks, half of the animals were sacrificed, and T cell responses analyzed. The other half received a booster immunization identical to the priming regimen. Blood was drawn at weeks 3, 6, and 18 and antibody responses were analyzed.

Since monoclonal antibodies directed against immune checkpoints were not applicable for the modulation of anti-Env antibody responses, we evaluated an alternative blocking system by intramuscular co-electroporation of DNA encoding the soluble ectodomains of PD-1 or PD-L1. To first confirm a sufficient duration of plasmid-driven expression of recombinant proteins after DNA electroporation, we administered a plasmid encoding luciferase and monitored the expression of this reporter enzyme (Figure 4A). Given the durable expression over a time-period of three weeks, we aimed to co-electroporate DNA encoding for the soluble ectodomains of murine PD-1 (sPD-1) or PD-L1 (sPD-L1) with HIV-1 DNA vaccines. To control plasmid-driven effects, an empty vector control (mock) was included. The animals were immunized in a prime-boost regimen by i.m. electroporation and cellular and humoral immune responses analyzed at different time-points (Figure 4B).

**Figure 5:**
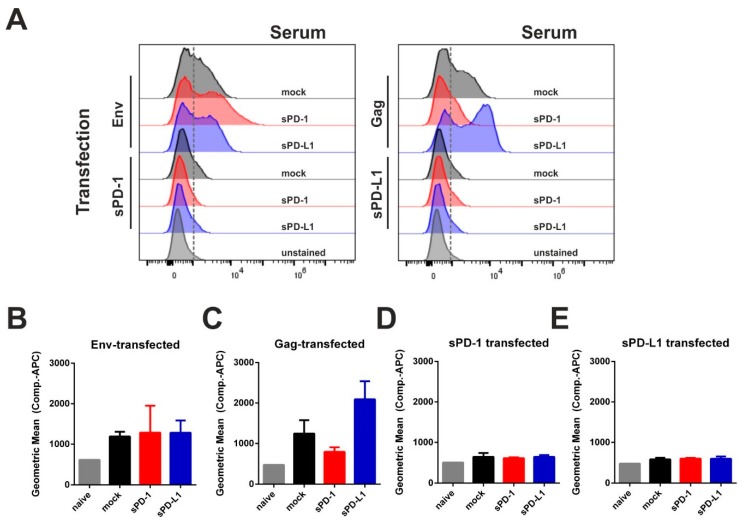
Soluble PD-1 and PD-L1 ectodomain co-expression is not inducing an autologous antibody response. 293T cells were transfected with plasmids encoding for Env, Gag, sPD-1, or sPD-L1. Twenty-four hours after transfection, cells were treated with Brefeldin A for 6 h in order to inhibit protein transport. Subsequently, cells were fixed, permeabilized, and incubated with sera from mice immunized with Env- and Gag-DNA either with empty vector (mock) or with corresponding checkpoint ectodomain DNA (sPD-1, sPD-L1). Murine antibodies were detected using an APC-conjugated anti-mouse IgG1 antibody. Shown are the histograms of transfected cells (**A**) as well as the Geometric Mean Fluorescence Intensity of Env- (**B**), Gag- (**C**), sPD-1 (**D**), and sPD-L1 transfected cells (**E**) after incubation with immunized mouse sera and the respective secondary antibody. (**A**) Data are representative of three independent experiments. (**B**–**E**) Data represent the mean with SEM of one out of three representative experiments with three sera samples from each group.

In order to analyze immunogenicity of the given ectodomains, we transfected 293T cells with each single plasmid used for immunization. In this regard, PD-1 and PD-L1 expression was validated by intracellular staining (Figure S6). The transfected cells were incubated with sera from DNA-immunized animals (see Figure 4) and a fluorophore-conjugated secondary anti-mouse IgG antibody was used for flow-cytometry evaluation (Figure 5A). Here we observed serum-derived antibodies against Env and Gag, but not against PD-1 or PD-L1 (Figure 5B–E). Since no serum antibodies specific for the soluble syngenic ectodomains were measurable, we concluded that in contrast to the monoclonal antibodies, DNA-based checkpoint inhibition did not elicit an anti-drug humoral response.

### 3.5. Co-Electroporation of PD-L1 Encoding DNA Modulates HIV-1-Specific Immune Responses

**Figure 6:**
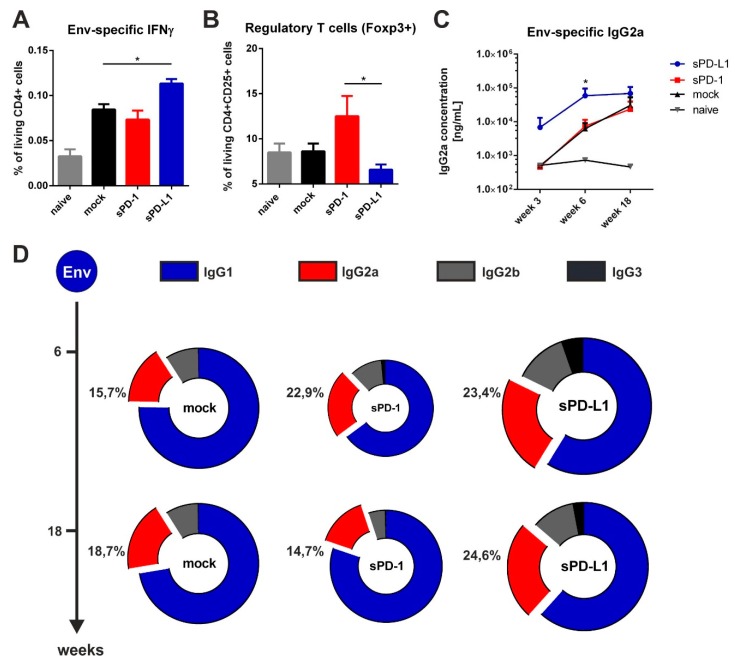
Soluble PD-L1 ectodomain co-expression enhances Env-specific immune responses. (**A**) Percentage of CD4+ T cells producing IFNγ after in vitro stimulation with HIV Env T helper peptide (measured by intracellular cytokine staining) in mice two weeks after immunization. Shown are mean values with SEM (*n* = 6) and significant differences between groups (one-way ANOVA analyses followed by Tukey’s multiple comparison test; * *p* < 0.05). (**B**) Frequency of regulatory T cells in the spleen of BALB/c mice 20 weeks after boosting. Shown are mean values with SEM of 18 animals from three independent experiments and significant differences between groups (one-way ANOVA analyses followed by Tukey’s multiple comparison test; * *p* < 0.05). (**C**) Quantitative Env-specific IgG2a antibody responses in the sera of BALB/c mice over a time-period of 18 weeks. Shown are mean values with SEM of 18 animals from three independent experiments and significant differences between groups (one-way ANOVA analyses followed by Tukey’s multiple comparison test; * *p* < 0.05). (**D**) Antibody subtype patterns of Env-immunized mice two (week 6) and 14 weeks (week 18) after the prime-booster immunization regimen. The ring size represents the overall antibody response. Shown are the mean percentages (*n* = 6) of each subtype based on the overall antibody response of a representative experiment (from three independent experiments). Each subtype was analyzed by ELISA with identical amounts of HRP-conjugated anti-mouse IgG1 (blue), IgG2a (red), IgG2b (gray), and IgG3 (black) antibodies.

By measuring antigen-specific CD4 T cell responses two weeks after priming (Figure 4), we observed a significant increase in Env-specific CD4 T cells secreting the T_H_1 cytokine IFNγ in mice co-electroporated with sPD-L1 DNA compared to the mock control (Figure 6A). We also evaluated the frequency of regulatory T cells in the spleen 20 weeks after the last immunization. On the one hand, we observed an increased expression of the transcription factor Foxp3 in sPD-1 co-electroporated animals. On the other hand, this expression was significantly downregulated after sPD-L1 co-electroporation (Figure 6B). When measuring the humoral immune response in these DNA-immunized mice, we observed a rapid induction of Env-specific IgG2a antibodies after priming. The serum levels of these antibodies was long-lasting and significantly higher compared to mock treated animals (Figure 6C). Interestingly, also IgG2b and IgG3 antibodies were durably enhanced by PD-L1 co-electroporation resulting in a more balanced Env-specific antibody subtype pattern (Figure 6D). In contrast to sPD-L1, sPD-1 did not elicit these effects and initially led to a decreased overall antibody response. Taken together, these data indicate that co-electroporation of DNA encoding the soluble ectodomain of PD-L1 together with HIV-1 DNA-based vaccine modulated vaccine-specific immune responses.

## 4. Discussion

In HIV-1 infected patients, humoral immune responses against Env protein are largely restricted to the IgG1 isotype [27,28,29,30]. Vaccination studies on mice demonstrated that the immune response against Env—in contrast to envelope proteins of other viruses—is strongly T_H_2-biased, which is partly caused by its unique glycosylation profile [11,12,22]. At the same time, T_H_1 associated Env-specific antibodies are correlating with an efficient virus control in elite controllers [1,3,7,31,32].

Application of suitable adjuvants in the vaccine formulation is a widely-used approach to modulate T-helper responses and subsequently the antibody subtype pattern [33]. In the context of HIV-1 Env vaccination, however, the addition of a plasmid encoding for IL-12, anti-mIL-10R Mab, or Alum did not significantly alter the IgG1 subtype response [34].

Previously we demonstrated a shift of the antibody response against Env towards T_H_1 IgG2a antibody production in mice by intrastructural help. However, this method requires preliminary induced T_H_1 immune responses against heterologous proteins [12,21,22].

Checkpoint inhibitors are widely used in the field of immunomodulation to treat melanoma and other cancers [16,17]. However, also in the context of HIV-1 infection, T cell exhaustion in chronically infected patients has been observed. This results in reduced T cell responses after enhanced expression of immune checkpoints [35,36,37]. Antiretroviral therapy can partly counteract virus-mediated T cell exhaustion, although immune checkpoint expression still remains increased compared to healthy individuals [38]. In vitro blockade of PD-1 in PBMCs of chronically infected patients led to the observation of improved HIV-specific T cell help to natural killer (NK) cell responses [39]. Additionally, the treatment of SIV-infected macaques with anti-PD-1 resulted in the expansion of virus-specific CD8 T cells with enhanced functionality [40]. These findings drew further interest towards the applicability of checkpoint inhibitors in the context of HIV-1 infection [41,42].

In this study, checkpoint blockade was applied after vaccination with VLPs containing Env and Gag or by i.m. electroporation of the respective plasmids. Therefore, CPIs were systemically administrated via intraperitoneal injections for a total time-period of two weeks. However, for both vaccination platforms no enhancement of HIV-specific immune responses by CPIs was observed. This could be due to native and adaptive immune responses induced by the treatment antibodies itself. Bernard-Tessier et al. reported an increased eosinophil count after treatment of patients with antibodies directed against PD-1 or its ligand PD-L1 while other immune cells remained unaffected [43]. Hypereosinophilia has also been discussed to be a parameter for the onset of immune-related adverse events (irAEs) [44]. These inflammatory side effects are associated with checkpoint inhibitor therapy [45]. Since eosinophils produce the T_H_2 cytokines IL-4 and IL-5 upon activation, we might have induced those irAEs in the mice after monoclonal antibody treatment resulting in the observed spontaneous secretion of IL-4 and IL-5 in the spleens of antibody-treated animals (Figure 3A,B). Furthermore, M2 macrophages could have been recruited towards the intraperitoneal injection site. Fox et al. showed that xenograft rejection by T cells is mediated by these infiltrating macrophages in the murine peritoneum [46]. Therefore, the application of the xenogenic antibodies via the intraperitoneal route might have been the cause of an innate immune response induced by the monoclonal antibodies to the checkpoint molecules, which resulted in the recruitment of immune cells towards the injection site.

Additionally, the elicitation of anti-drug antibodies was observed (Figure 3D,E). This induction was only detectable in animals receiving CPIs. A T cell-dependent B cell response can be locally triggered in the peritoneum, as demonstrated by Rangel-Moreno et al. [25]. Therefore, the blockade of PD-1 and its ligands might result in the induction of an adaptive immune response directed against the drugs themselves.

These anti-drug antibodies induced by systemic checkpoint inhibitor administration might strongly interfere with the HIV-1-specific immune responses. Therefore, we locally blocked immune checkpoints by co-electroporation of soluble PD-1 and PD-L1 ectodomains. It has been shown that sPD-1 is able to block immune checkpoint interactions in vivo [47,48]. However, also sPD-L1 serves as a receptor antagonist for the inhibitory activity of transmembrane PD-L1 [49]. Since these soluble ectodomains are autologous (murine), no anti-drug responses were detected after co-electroporation (Figure 5).

Strikingly, of the used soluble ectodomains only sPD-L1 improved HIV-1 Env specific immune responses. An explanation could be the respective binding partners of the soluble immune checkpoints. Whereas PD-1 is capable of binding both PD-L1 and PD-L2, PD-L1 is not only able to interact with the inhibitory receptor PD-1, but also with CD80. This interaction—which can also occur on the same cell—has been shown to be important for the induction of optimal T cell responses [50]. Additionally, sPD-L1 can bind to the constitutively expressed PD-1 on regulatory T cells. Therefore, it might diminish the capacity of regulatory T cells to inhibit T cell proliferation and cytokine production [51]. This is concomitant with the reduced frequency of regulatory T cells in mice co-electroporated with PD-L1.

Since PD-L1 and PD-1 expression varies on different cell types during the germinal center reaction, not only CD4 T cells might be responsible for the observed effects mediated by soluble PD-L1. Direct effects of the checkpoint inhibitors on B cells or transient populations of T follicular helper cells are not to be excluded. Adoptive transfer of both CD4 and B cells from primed mice at various time-points could be done to further elucidate the underlying immunological mechanism.

## 5. Conclusions

In this study, we demonstrated that co-electroporation of the soluble ectodomain of PD-L1 together with HIV-1 antigens enhanced and modulated HIV-1 specific immune responses. Whether this platform can be applied on other DNA-based vaccines needs to be validated in subsequent experimental trials. Nevertheless, i.m. electroporation of soluble checkpoint ectodomains represents an inexpensive and effective platform that might be beneficial in eliciting potent immune responses.

## Supplementary Materials

The following are available online at https://www.mdpi.com/2076-393X/8/1/27/s1, Figure S1: Antigen and antibody production for quantitative ELISA; Figure S2: Virus-like particle size and homogeneity; Figure S3: Env-specific CD4 T cell responses; Figure S4: Gag-specific CD4 T cell responses; Figure S5: Antigen-specific TH2 cytokine production; Figure S6: Expression of soluble ectodomains.
