# Supplementary material for: Modulation of Vaccine-Induced HIV-1-Specific Immune Responses by Co-Electroporation of PD-L1 Encoding DNA
Source: Vaccines (Basel). 2020 Jan 14;8(1):27. doi: 10.3390/vaccines8010027 (PMC7157229; doi:10.3390/vaccines8010027)
Supplement: Supplementary file 1 [file vaccines-08-00027-s001.pdf]

Supplementary Materials:

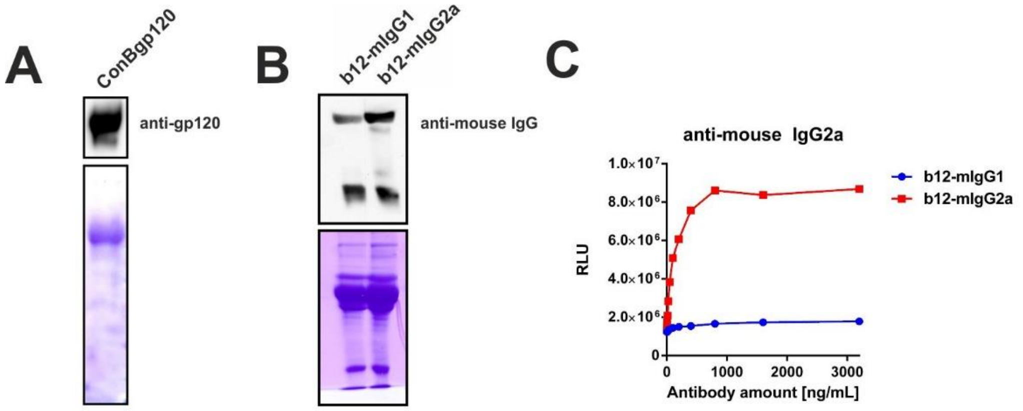

Figure S1. Antigen and antibody production for quantitative ELISA. (**A**) ConBgp120 (coating antigen) produced in 293F cells with quality and purity validated by Western Blot and Coomassie staining. (**B**) Monoclonal b12 antibody production with murine heavy chains (antibody standard) in 293T cells. Quality and purity was validated by Western Blot and Coomassie staining. (**C**) Specificity of the produced b12-mIgG2a antibody towards the respective HRP-conjugated anti-mouse IgG2a antibody in a dosage-dependent manner.

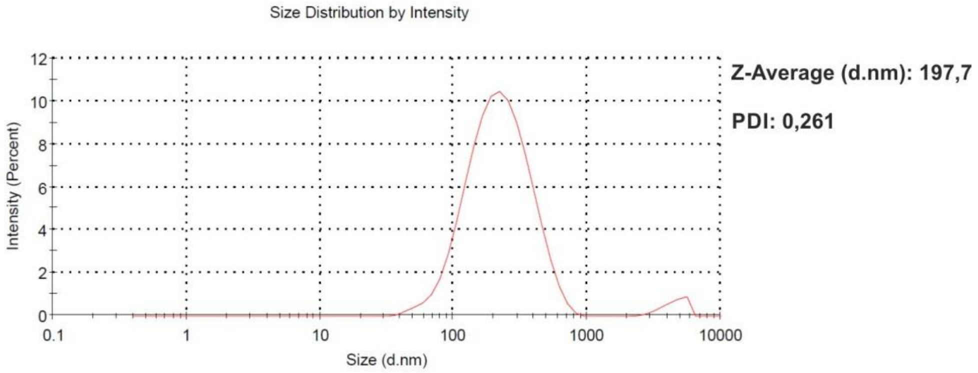

Figure S2. Virus-like particle size and homogeneity. VLPs produced in 293T cells and purified over a sucrose cushion containing Env (pConBgp140-GCD) and Gag (Hgpsyn) were diluted in water (1:4000) and measured by ZetaSizer Nano S90 (Malvern Pananalytical, Kassel). Shown are the dynamic size and the particle dispersity index (PDI) of the measured particles.

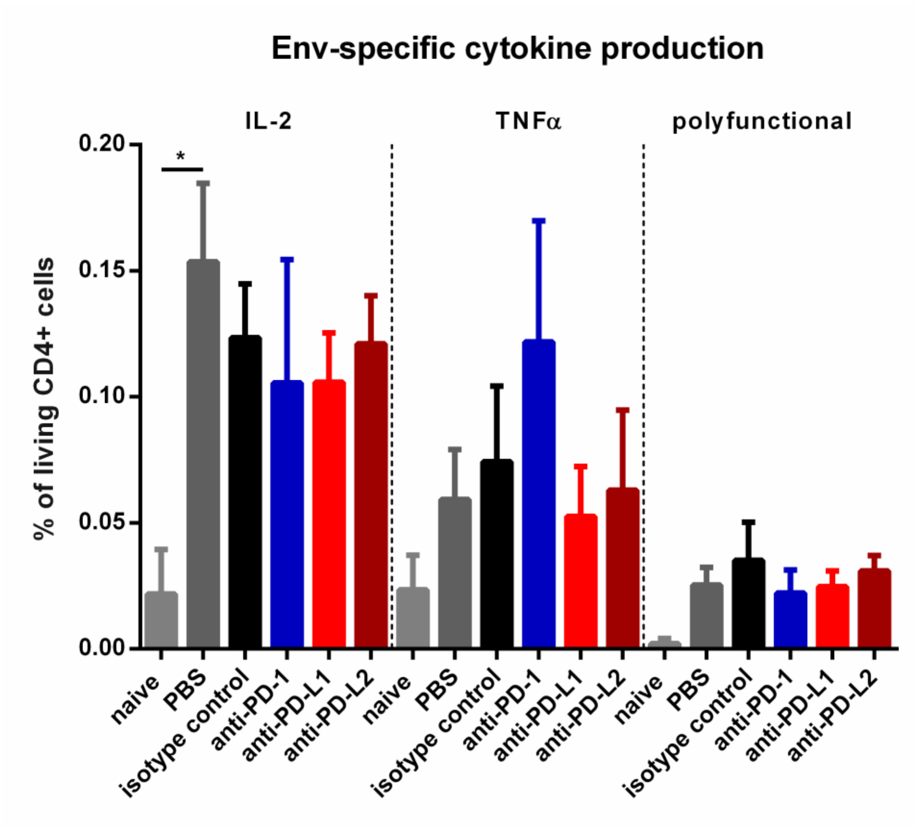

Figure S3. Env-specific CD4 T cell responses. Living Env-specific CD4 T cells secreting IL-2, TNFα or those cytokines simultaneously with IFNgamma (polyfunctional) in the spleen of BALB/c mice two weeks after immunization. Shown are mean values with SEM (n = 4) and significant differences between the groups (one-way ANOVA analyses followed by Tukeys multiple comparison test; * $p <$ 0.05).

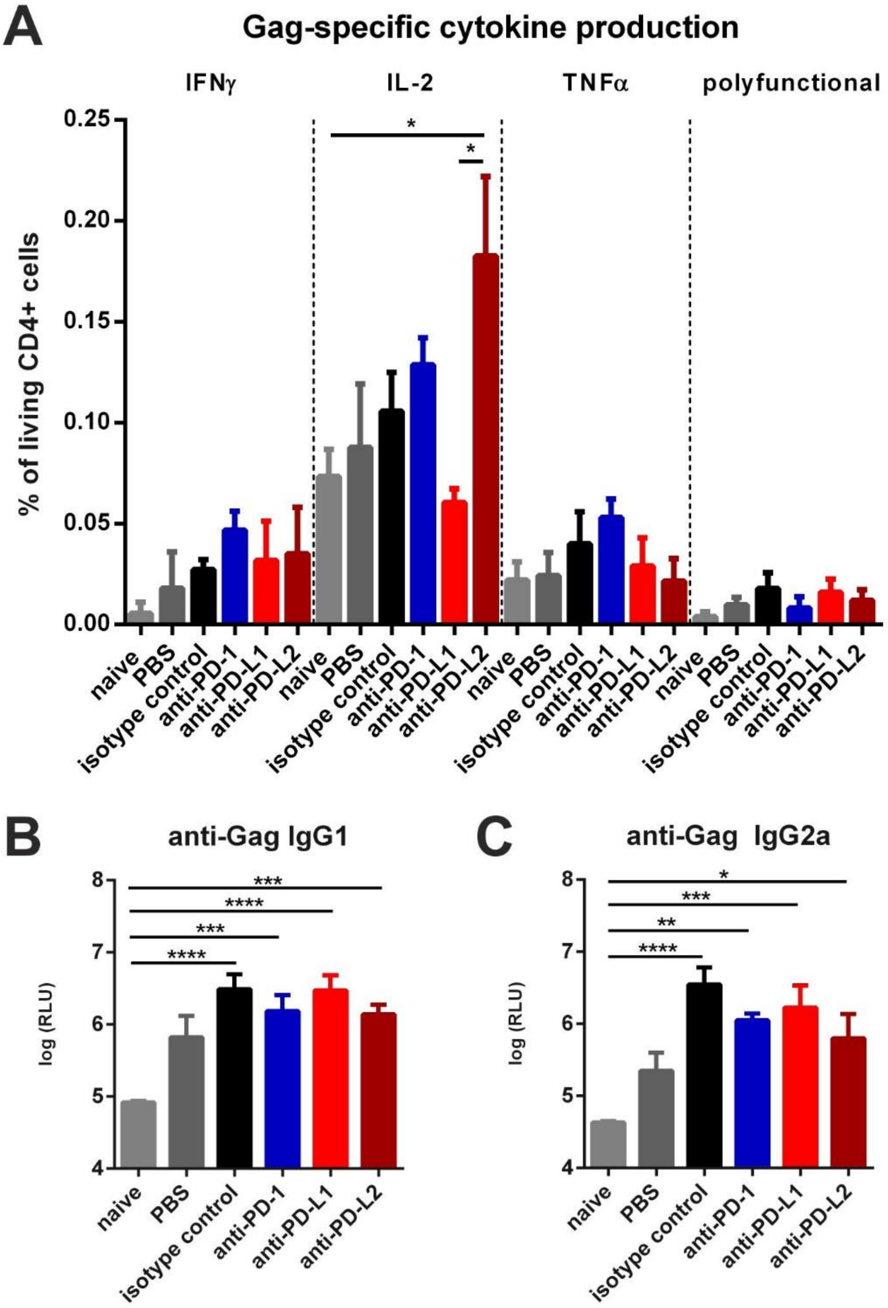

Figure S4. Gag-specific CD4 T cell responses. (**A**) Living Gag-specific CD4 T cells secreting IFNγ, IL-2, TNFα or all three cytokines simultaneously (polyfunctional) in the spleen of BALB/c mice two weeks after immunization. Shown are mean values with SEM (n = 4) and significant differences between the groups (one-way ANOVA analyses followed by Tukeys multiple comparison test; * $p < 0.05$). Gag-specific IgG1 (**B**) and IgG2a (**C**) antibody responses in the sera of BALB/c mice six weeks after priming. Shown are mean values with SEM (n = 4–6) and significant differences between the groups (one-way ANOVA analyses followed by Tukeys multiple comparison test; *$p < 0.05$, **$p < 0.01$, *** $p < 0.001$, **** $p < 0.0001$).

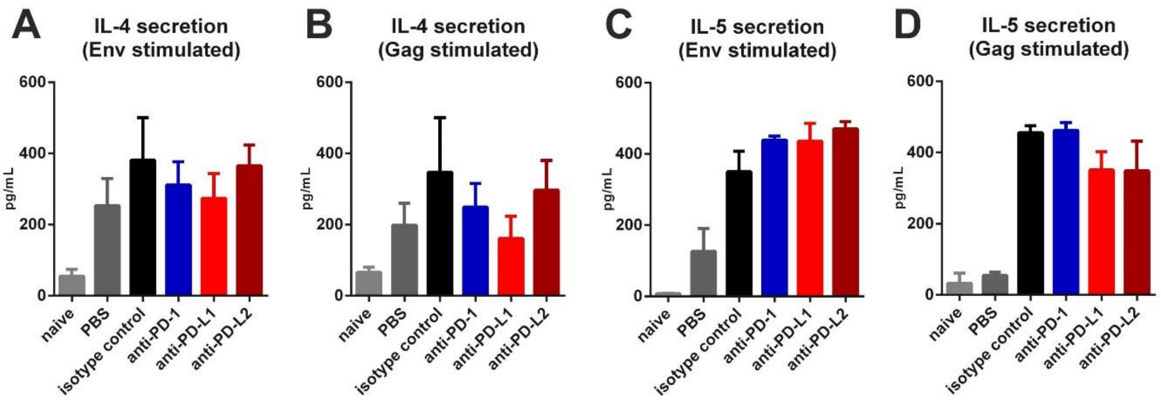

Figure S5. Antigen-specific TH2 cytokine production. Env- and Gag-specific secretion of IL-4 (**A**,**B**) and IL-5 (**C**,**D**) after three-day culture of splenocytes of BALB/c mice two weeks after immunization and two days after the last CPI treatment. Shown are mean values with SEM (*n* = 2–4), as depicted partly in Fig. 3C (naïve and PBS).

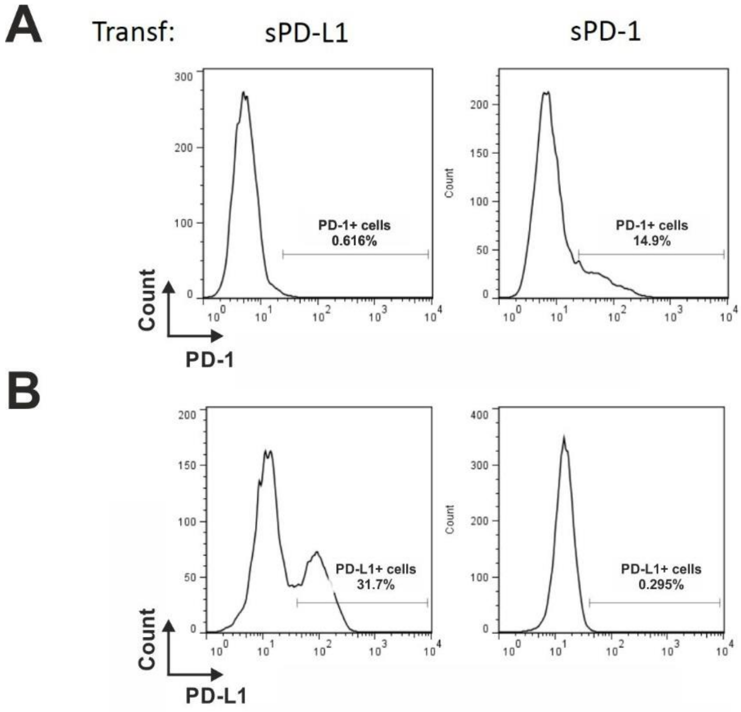

Figure S6. Expression of soluble ectodomains. 293T cells were transfected with the expression plasmids encoding for sPD-1 or sPD-L1. Cells were fixed and permeabilized. PD-1 expression was validated with an anti-PD-1 PE-Cy7 antibody (**A**), PD-L1 expression could be detected by Rat-anti-PD-L1 and subsequent anti-Rat IgG FITC antibody staining (**B**).
